# Electronic Band Structure and Optical Properties of HgPS_3_ Crystal and Layers

**DOI:** 10.1021/acs.jpcc.4c00562

**Published:** 2024-05-24

**Authors:** Beatriz de Simoni, Miłosz Rybak, Nikolas Antonatos, Artur P. Herman, Karolina Ciesiołkiewicz, Agata K. Tołłoczko, Maciej Peter, Adrianna Piejko, Kseniia Mosina, Zdeněk Sofer, Robert Kudrawiec

**Affiliations:** † Department of Semiconductor Materials Engineering, Wroclaw University of Science and Technology, Wybrzeże Wyspiańskiego 27, 50-370 Wrocław, Poland; ‡ Department of Inorganic Chemistry, University of Chemistry and Technology, 5 Technická, 166 28 Prague 6 - Dejvice, Czech Republic; § Department of Nanometrology, Wroclaw University of Science and Technology, Janiszewskiego 11/17, 50-370 Wrocław, Poland

## Abstract

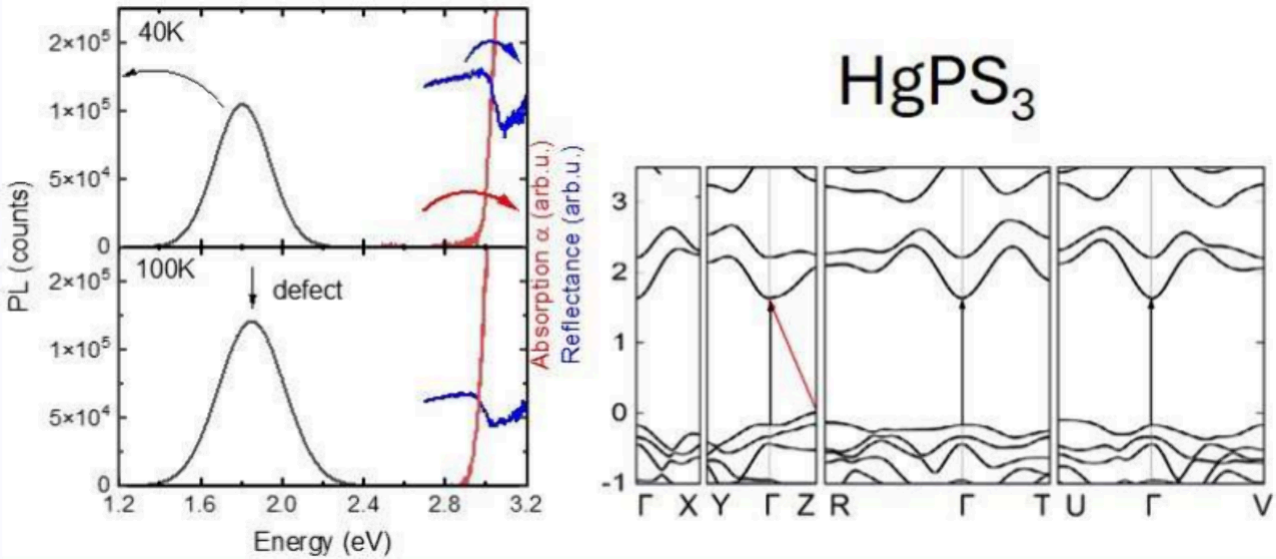

Transition metal thiophosphates (MPS_3_) are of great interest due to their layered structure and magnetic properties. Although HgPS_3_ may not exhibit magnetic properties, its uniqueness lies in its triclinic crystal structure and in the substantial mass of mercury, rendering it a compelling subject for exploration in terms of fundamental properties. In this work, we present comprehensive experimental and theoretical studies of the electronic band structure and optical properties for the HgPS_3_ crystal and mechanically exfoliated layers from a solid crystal. Based on absorption, reflectance and photoluminescence measurements supported by theoretical calculations, it is shown that the HgPS_3_ crystal has an indirect gap of 2.68 eV at room temperature. The direct gap is identified at the Γ point of the Brillouin zone (BZ) ≈ 50 meV above the indirect gap. The optical transition at the Γ point is forbidden due to selection rules, but the oscillator strength near the Γ point increases rapidly and therefore the direct optical transitions are visible in the reflectance spectra approximately at 60–120 meV above the absorption edge, across the temperature range of 40 to 300 K. The indirect nature of the bandgap and the selection rules for Γ point contribute to the absence of near-bandgap emission in HgPS_3_. Consequently, the photoluminescence spectrum is primarily governed by defect-related emission. The electronic band structure of HgPS_3_ undergoes significant changes when the crystal thickness is reduced to tri- and bilayers, resulting in a direct bandgap. Interestingly, in the monolayer regime, the fundamental transition is again indirect. The layered structure of the HgPS_3_ crystal was confirmed by scanning electron microscopy (SEM) and by mechanical exfoliation.

## 1 Introduction

The scientific interest in layered transition-metal dichalcogenides (TMD) owing to their outstanding electronic, optical, structural, and magnetic properties dates back to the 1970s.^1−4^ Nevertheless, the discovery of atomically thin graphene^5^ in the early 2000s gave rise to a great deal of attention devoted to two-dimensional (2D) TMDs and their unique properties.^6^ They are compounds with MX_2_ formula, where M is a transition metal and X is a chalcogen (X = S, Se, or Te), forming a 3-atom-thick structure of one M atom sandwiched between two X atoms. Among many others, one of the main reasons for the interest in TMDs is the tuning of bandgap according to the thickness of the material: while the bulk form has an indirect bandgap, their monolayer counterpart has a direct gap^7−11^, enabling them to find potential use in a plethora of applications, such as in optoelectronics and valleytronics^12−14^. Other layered semiconducting compounds such as alpha lead oxide (α-PbO) also exhibit indirect–direct gap crossover when going from multilayer to monolayer.^15,16^

Another family of materials whose scientific interest has been growing in the past years is the transition-metal phosphorus trichalcogenides (TMPTs). Closely related to TMDs, these compounds possess the formula MPX_3_, where M is a transition metal, a divalent cation, and X is commonly Se or S. One remarkable advantage of TMPTs over TMDs is the fact that they can have larger bandgaps than TMDs, which are limited to around 2 eV,^17^ while on TMPTs they range from 1.3 to 3.5 eV,^18,19^ enhancing their efficiency for absorbing light; bulk HgPS_3_, for example, has a bandgap of approximately 2.7 eV at ambient conditions.^20^ The bandgap of α-PbO can reach up to 3.3 eV in the monolayer limit.^21^

While all the materials belonging to the MPS_3_ family crystallize in a monoclinic *C*2/*m* structure at ambient conditions, HgPS_3_ is unique as it is the sole member that obtains a triclinic *P*1̅ structure^22,23^. Remarkably, even the selenide counterpart HgPSe_3_ possesses a monoclinic structure (space group *C*2/*c*), distinguishing HgPS_3_ from the others. Another significant difference, which is also true in the case of HgPSe_3_, is the (distorted) tetrahedral coordination between the metal and chalcogen, while it is octahedral for all the other members of the family.^24^ Such a distinctive deviation of HgPS_3_ with respect to its crystal structure renders the material quite unique and appealing. However, reports in the literature are scarce. Calareso et al. have reported the reflectivity, absorption, and X-ray photoelectron spectroscopy in room temperature, measuring a forbidden direct bandgap of 2.67 eV.^20,25^ Nonetheless, this was concluded solely by experimental observations because the band structure of such a compound had not been established yet. Hence, further investigation into the properties of HgPS_3_ are sought after. In this report, by combining emission-like spectroscopy (photoluminescence) with absorption and reflectivity, and interpreting these results on the basis of state-of-the-art first-principles calculations, we unambiguously discern the observed optical transitions. The electronic band structure of bulk and few-layer HgPS_3_ is presented, based on density-functional theory (DFT) calculations combined with optical measurements.

## 2 Experimental Section

### 2.1 HgPS_3_ Synthesis

HgPS_3_ was synthesized by placing mercury (99.999%, Strem, Germany), red phosphorus (99.999%, Strem, Germany), and sulfur (99.999%, Strem, Germany) in a quartz ampule in stoichiometric quantities to a total amount of 15 g together with 0.25 g of HgI_2_ acting as a transport agent. The ampule was melt-sealed with an oxygen–hydrogen flame at a pressure of 1 × 10^–3^ Pa. Liquid nitrogen was employed to prevent mercury evaporation during the sealing process. The ampule was then placed in a crucible furnace and heated at 400 °C while the cold end was kept below 200 °C for 48 h. Afterward, the ampule was moved to a horizontal two-zone furnace where the growth zone was heated at 450 °C while the reaction mixture was kept at 350 °C for 24 h. Then, the gradient was reversed, and the changed zone was kept at 400 °C while the growth zone decreased from 350 to 300 °C in the course of 5 days. Finally, the ampule was opened inside an argon glovebox.

### 2.2 Structural and Morphological Characterization

The X-ray diffraction (XRD) pattern was acquired using a Bruker D8 Discoverer powder diffractometer (Bruker, Germany) in Bragg–Brentano parafocusing geometry and applying Cu Kα radiation (λ = 0.15418 nm, *U* = 40 kV, *I* = 40 mA). The diffraction patterns were collected between 5° and 90° of 2θ with a step size of 0.020°, and the acquired data were evaluated using HighScore Plus 3.0e. The XRD reflections were simulated by Crystal Diffract software. The morphology of bulk HgPS_3_ was investigated via scanning electron microscopy (SEM) images through a field emission gun electron source (Tescan Lyra dual microscope) and elemental composition and mapping of the materials were obtained by an energy dispersive spectroscopy (EDS) analyzer (X-MaxN) with a 20 mm^2^ SDD detector (Oxford Instruments) and AZtecEnergy software. The sample was placed on a carbon conductive tape. SEM and EDS measurements were carried out using an electron beam in the range 5–10 kV. The grid was Cu (200 mesh; Formvar/carbon). X-ray photoelectron spectroscopy (XPS) measurements were performed on an ESCAProbeP spectrometer (Omicron Nanotechnology Ltd., Germany) employing a monochromatic aluminum X-ray radiation source (1486.7 eV). Wide-scan surveys of all elements were performed with subsequent high-resolution scans of mercury (Hg 4f), phosphorus (P 2p), and sulfur (S 2p). The samples were placed on a silicon wafer. An electron gun (1–5 V) was utilized to eliminate the sample charging during measurement. All XPS survey spectra were afterward analyzed by CasaXPS software.

### 2.3 Optical Characterization

HgPS_3_ crystals were characterized by temperature-dependent absorption and reflectance in the ranges 10–300 and 40–300 K, respectively. For accomplishing this, samples were placed into a cryostat at vacuum conditions (≈10^–6^ bar) and illuminated with white light from a halogen lamp, and the reflected light (or transmitted in the case of absorption) was dispersed with a grating monochromator. For both experiments, the light was chopped at 280 Hz and the signal was measured by lock-in technique. For photoluminescence (PL) and Raman measurements, a monochromator with a multichannel liquid nitrogen cooled Si CCD array detector was employed. The sample was excited by a 405 nm laser line for PL and 532 nm for Raman, with power of a few microwatts for PL and 100–200 μW for Raman. Raman measurements were performed in backscattering configuration. Power-dependent PL was collected at 10 and 150 K. A long working distance 50× objective was used to properly focus on the samples that were kept in vacuum inside a coldfinger, which allows measurements in the range 10–300 K.

### 2.4 Computational Details

The DFT calculations have been performed in the Vienna Ab Initio Simulation Package (VASP).^26^ The electron–ion interaction was modeled using projector-augmented-wave technique.^27^ The Perdew–Burke–Ernzerhof (PBE) exchange–correlation (XC) functional was employed.^28^ A plane-wave basis cutoff of 500 eV and a 12 × 12 × 8 Monkhorst–Pack k-point grid for BZ integrations were set^29^ (except for vacuum systems when it was 12 × 12 × 1). These values assured the convergence of the lattice constants and the electronic gaps were within precision of 0.001 Å and 0.001 eV, in relation to the convergence due to the k-point grid and the cutoff energy, respectively. A Gaussian smearing of 0.02 eV was used for integration in reciprocal space. The semiempirical Grimme’s correction with Becke–Johnson damping (D3-BJ) was employed to properly describe the weak van der Waals (vdW) forces.^30^ The spin–orbit (SO) interaction was taken into account, also at the level of stress-tensor geometry optimization (with a 0.005 eV/Å force criterion and 0.1 kbar stress one). For single layers, a vacuum of the thickness of 20 Å was added to mimic the isolated system. The density-functional-perturbation theory was used to calculate the phonon frequencies at the Γ point of the Brillouin zone.^31^

## 3 Results and Discussion

### 3.1 Material Characterization

**Figure 1:**
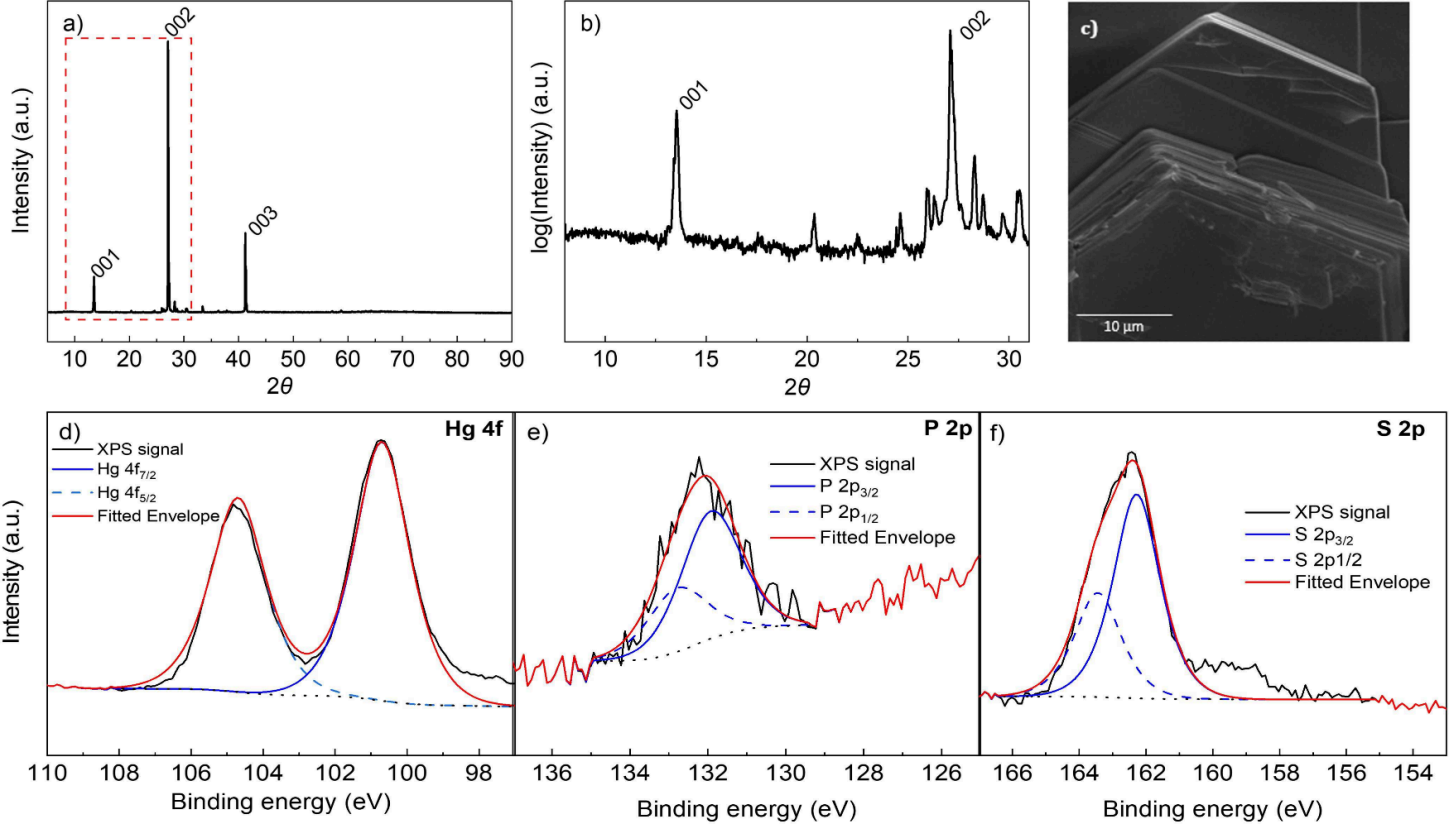
Structural characterization of HgPS_3_. a) XRD pattern with the most intense peaks labeled, b) the enlarged area marked with a red dashed frame from a). c) SEM image. High-resolution XPS spectra of d) Hg 4f, e) P 2p, and f) S 2p.

**Scheme 1:**
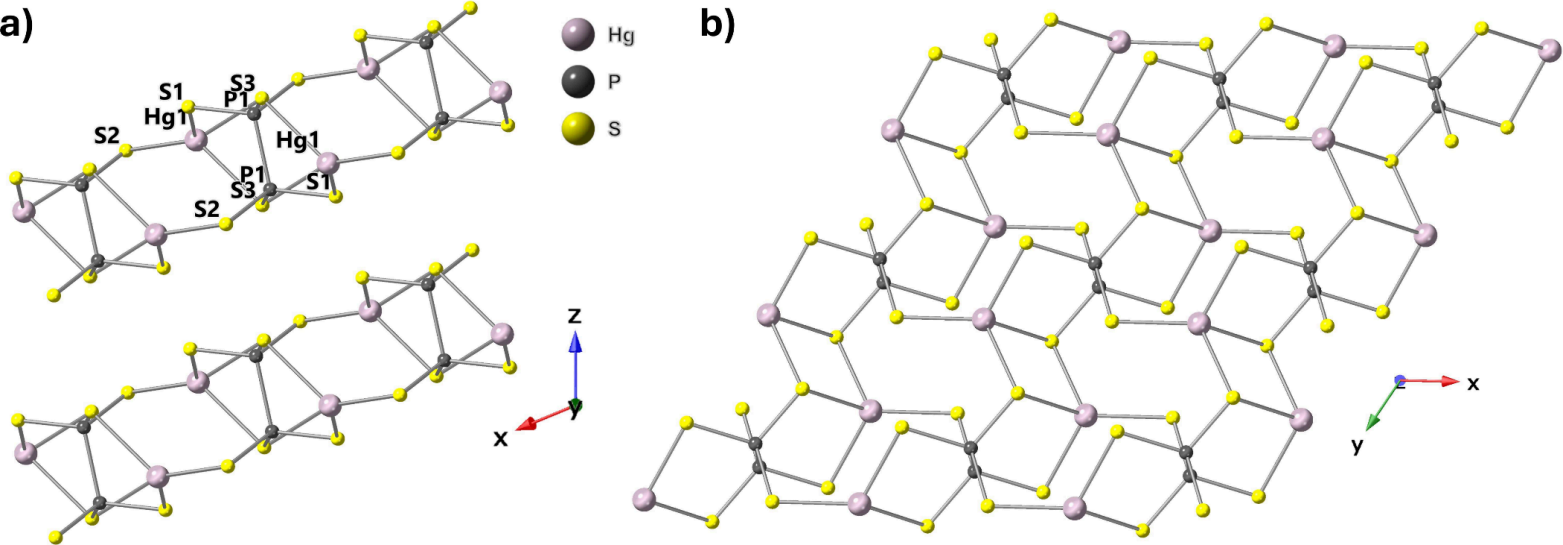
Crystal Structure of HgPS_3_: a) Side and b) Top Views

The structural characterization of HgPS_3_ was performed through XRD. There are no experimental reports about the diffraction pattern of the material in the literaturem and no record could be found in the database. However, by simulating the reflections of the bulk material through CrystalDiffract software, we managed to confirm the triclinic structure of HgPS_3_ belonging to the *P*1̅ space group. The triclinic structure of HgPS_3_ is illustrated in Scheme 1. A double layer parallel to the *a*–*b* plane is formed where the Hg atom is coordinated tetrahedrally to four S atoms to form distorted HgS_4_ tetrahedrons. Two P atoms are bonded, and each part of the pair is bonded to three S atoms to form a (P_2_S_6_)^4–^ group. HgPS_3_ layers are held together by weak van der Waals forces constituting the material as layered. The geometric parameters of the structure of HgPS_3_ are listed in Table 1. The sharp diffraction peaks are an indication of the high crystallinity of the material with a preferred orientation along the (00*L*) direction, as evidenced by the indexing of the peaks (Figure 1a). The simulated reflections are in good agreement with the experimental data, as shown in Figure S1. The peaks in the red dashed frame of Figure 1a are shown in closer detail in Figure 1b, and the intensity is plotted in logarithmic scale to reveal the presence of more reflections with lower intensity. This is to highlight the polycrystallinity of the material. The SEM image in Figure 1c illustrates the layered nature of the material, and the EDS analysis confirmed the 1:1:3 stoichiometry of HgPS_3_ (Figure S2) and the uniform distribution of all three elements across the material (Figure S3). In addition, the surface composition of HgPS_3_ was investigated through XPS. The wide-survey spectrum confirmed the expected presence of mercury, phosphorus, and sulfur including adventitious carbon and oxygen (Figure S4). The high-resolution XPS spectrum of Hg 4f shows two well-defined peaks corresponding to Hg 4f_7/2_ and 4f_5/2_ with a 4.0 eV spin–orbit separation (Figure 1d). The high-resolution spectra of P 2p and S 2p depict a peak split into P 2p_1/2_ and P 2p_3/2_ with a peak separation of 0.87 eV (Figure 1e) and S 2p_1/2_ and S 2p_3/2_ with a peak separation of 1.16 eV (Figure 1f), respectively. The absence of oxidized species in all three high-resolution spectra is a fine indication of the material’s stability in air. Fourier transform infrared spectroscopy (FTIR) measurements were performed on an iS50R FTIR spectrometer (Thermo Scientific, USA). The measurement was performed by a diamond ATR crystal, DLaTGS detector, and KBr beamsplitter in the range 4000–400 cm^–1^ at a resolution of 4 cm^–1^. The measurement was performed in reflectance mode using a Smart SAGA reflectance accessory, a MCTD* detector, and a KBr beamsplitter. Figure S5 depicts the FTIR spectrum of HgPS_3_ showcasing three prominent peaks at 449, 598, and 1073 cm^–1^. In a similar fashion to Raman spectroscopy, there are no reports available, but we can compare our spectrum to other phosphorus-based materials. While the 450 cm^–1^ band is attributed to P–P vibrations,^32^ the intense peak located at 597 cm^–1^ is consistent with every MPS_3_ sample assigned to the P–S stretching vibrations.^33^ Finally, the broad peak at 1069 cm^–1^ corresponds to the phosphate bands due to partial oxidation of the surface of the material.^34^ Raman spectroscopy was performed in bulk (10–300 K) and exfoliated samples (300 K) without significant differences between them and the results are presented in Figures S7 and S8.

**Table 1:** Overview of HgPS_3_ Crystals Structure Parameters^23^,^a^

Lattice Parameters	Bond length (Å)
*a* = 6.252(3) Å	Hg–S1 = 2.554(8)
*b* = 6.262(4) Å	Hg–S2 = 2.439(9)
*c* = 7.126(6) Å	Hg–S3 = 2.643(8)
α = 96.21(6)°	P–S1 = 2.028(12)
β = 105.69(6)°	P–S2 = 2.036(11)
γ = 119.15(4)°	P–S3 = 2.036(11)
Cell Volume = 224.8 Å^–3^	P–P = 2.267(11)

a The atoms are labeled in Scheme 1a.

### 3.2 Optical Properties of HgPS_3_

**Figure 2:**
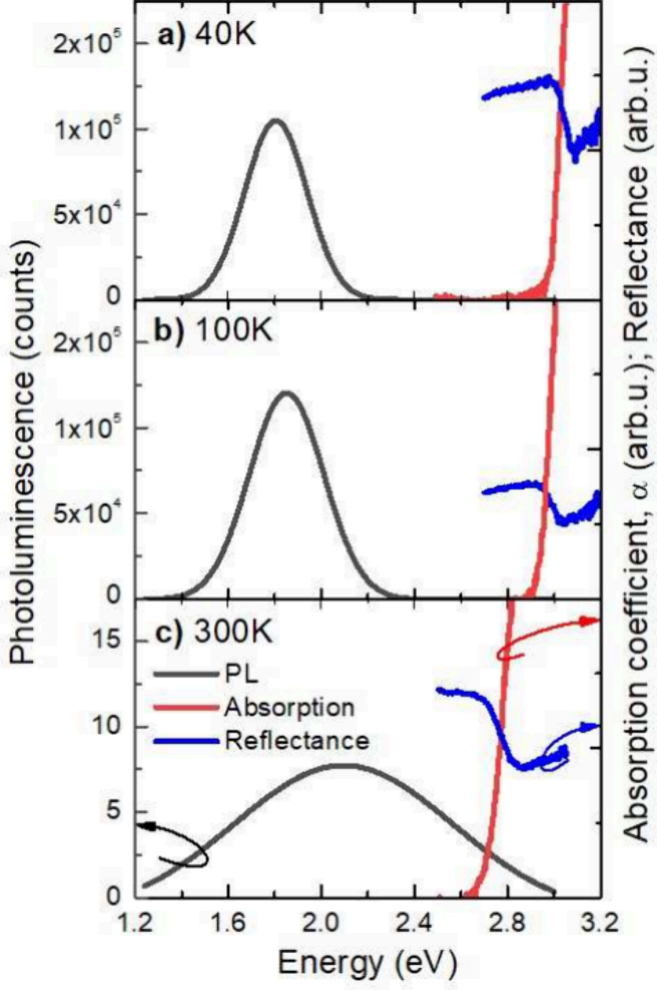
Direct comparison between photoluminescence (black), reflectance (blue), and absorption (red) spectra at a) 40 K, b) 100 K, and c) 300 K.

Figure 2 shows the comparison between absorption (red lines), reflectance (blue lines), and photoluminescence (black lines) spectra at selected temperatures. Different trends can be noted: the absorption starts a little below the reflectance resonance and together they move to lower energies with increasing temperature, while the PL peak moves the opposite way, and becomes weaker and broader from the low temperature regime to room temperature. The Stokes shift of ≈1.15 eV at 40 K between PL and absorption indicates that we are dealing with a defect-related emission. The fact that no emission is observed in the vicinity of the absorption edge can be treated as an indication of the indirect nature of the bandgap of HgPS_3_. This conclusion is consistent with^20^ our theoretical calculations of the electronic band structure presented in the following section, where the calculations of the oscillator strength for the direct optical transition at the Γ point of the Brillouin zone show that this strength is close to zero but significantly increases out of the Γ point. This suggests that for a perfect crystal the oscillator strength for an excitonic transition at the Γ point will be very weak, but in a real crystal such a transition can be observed, especially since a large oscillator strength appears near the Γ point. This means that emission precisely at the Γ point is not allowed but absorption near the Γ is allowed. Therefore, the resonance observed in the reflectance spectra is attributed to a free exciton (FX)-like absorption near the Γ point of BZ. The FX-like transition is not visible in the absorption spectra due to the large thickness of the sample (≈100 μm), which in this case makes it possible to determine the indirect gap. In Figure S10 we extend the discussion about oscillator strength by showing the energy difference among the three valence bands and two conduction bands closest to the bandgap, and the oscillator strength of each of these direct transitions. In addition, we show the single particle dielectric function for HgPS_3_ in Figure S11.

**Figure 3:**
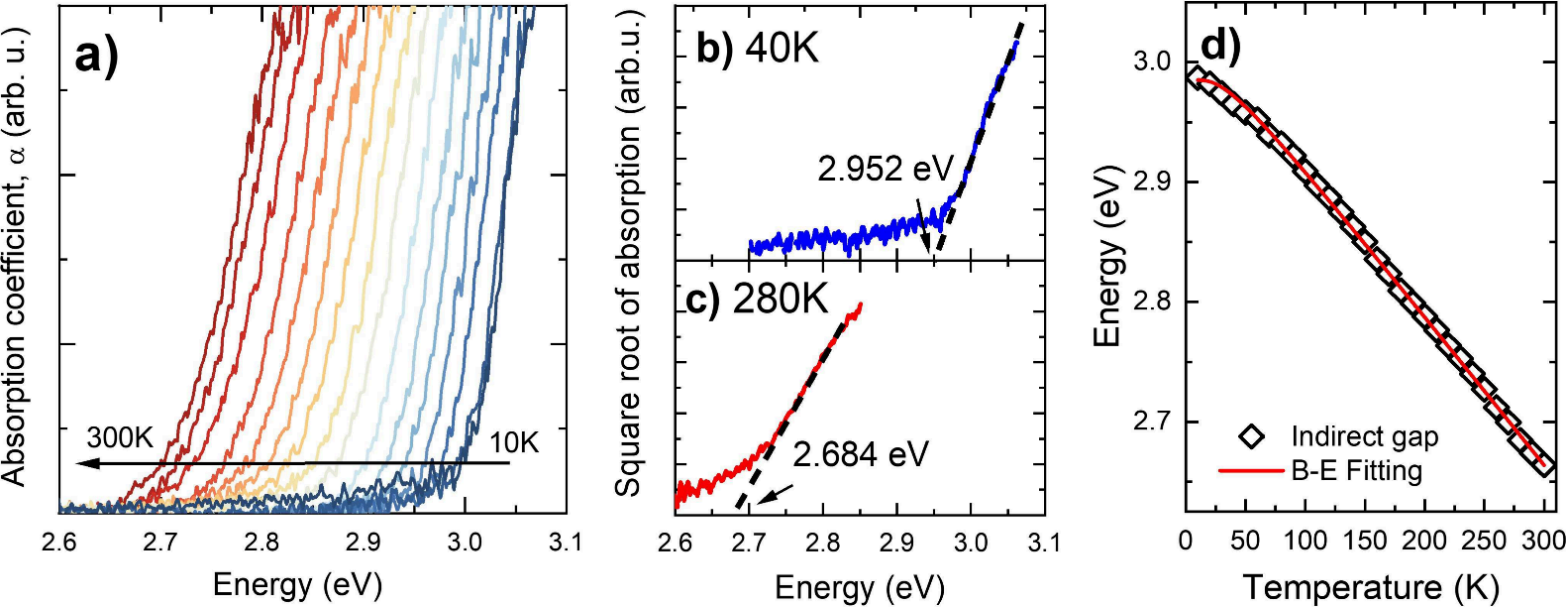
a) Evolution of absorption coefficient from 10 to 300 K. Lines are displayed at intervals of 20 K for better visualization. Tauc plots for extraction of allowed indirect gap energies *E*_*i*_ at b) 40 K and c) 280 K, given by . d) The acquired energies reveal a strong red shift of Δ*E*_*i*_ = 0.323 eV from 10 to 300 K.

The absorption spectra obtained from 10 to 300 K are presented in Figure 3a, and the first remark is that the absorption edge systematically shifts toward lower energies as the temperature rises. By means of Tauc plots, one can extract both forbidden direct (as suggested in ref (20)) and allowed indirect gaps, from linear extrapolation of the curves α^2/3^ × *E* and , respectively,^35^ and the difference between the acquired energies is rather negligible because of very similar exponents. Two examples can be seen in Figure 3b,c, in which Tauc plots for allowed indirect gap energies  and the extrapolation lines are shown for *T* = 40 and 280 K. The spectral position of the reflectance resonance in relation to the absorption edge led to the conclusion that the bandgap gap is rather indirect. Figure 3d shows the indirect gap energies obtained at each temperature and it reveals a strong red shift of Δ*E*_*i*_ = 0.323 eV from 10 to 300 K. The fact that the absorption edge does not show an excitonic peak is due to the large sample thickness as well as the contribution of phonons, which gets stronger with the rise in temperature since the number of phonons *n*_*q*_ follows Bose–Einstein statistics,
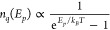
1where *E*_*p*_, *k*_*B*_, and *T* are the phonon energy, Boltzmann constant, and temperature, respectively.^36^

**Figure 4:**
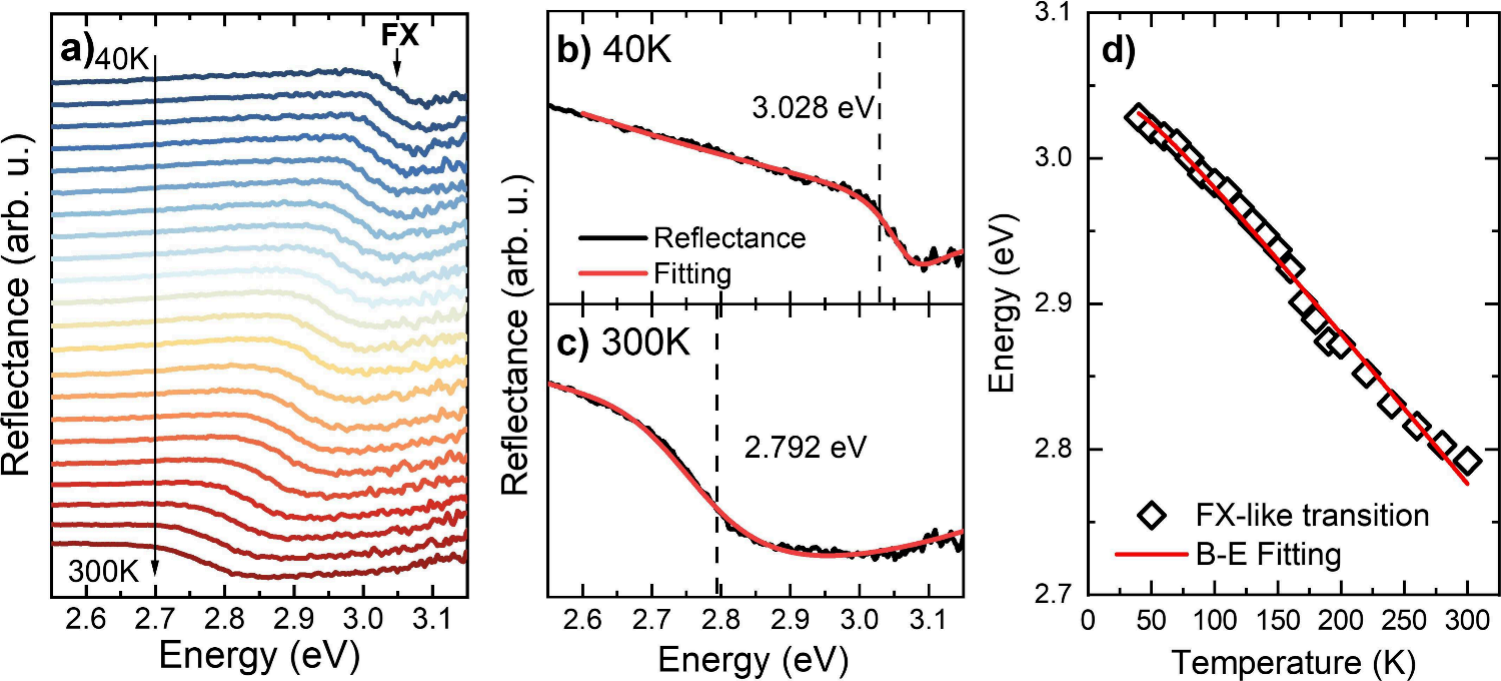
a) Reflectance spectra of HgPS_3_ from 40 to 300 K, in steps of 10 K from 40 and 200 K, followed by 20 K steps. Spectra are shifted vertically for better visualization. Equation 2 fitted to the reflectance spectrum at b) 40 K and at c) 300 K. d) Equation 3 (red line) fitted to FX energy (parameter *hν*_*x*_ from eq 2) at each *T*.

To identify the direct optical transitions, temperature-dependent reflectance was carried out from 40 to 300 K, and the results are depicted in Figure 4a. Only one feature was observed in the spectra: a resonance that we attribute to an FX-like transition near the Γ point of BZ. In general, the line shape of this resonance does not change throughout the temperature range, apart from the red shift and an increase of broadening with increasing temperature. In order to extract the spectral position of the resonance, the expression
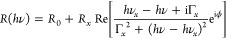
2was fitted to the reflectance spectra. *R*(*hν*) expresses the dependence of the reflectivity on the energy *hν*, *R*_0_ is a background, *R*_*x*_ is an amplitude, *hν*_*x*_ is the energy related to the direct transition (excitonic energy), Γ_*x*_ is the broadening parameter, and ϕ is a phase.^37^Figure 4 panels b and c show eq 2 fitted to the data at 40 and 300 K. The temperature dependence of the excitonic energy (parameter *hν*_*x*_ from eq 2) can be fitted by an expression that takes into consideration that the change in bandgap with temperature (*E*(*T*)) is proportional to the number of phonons *n*_*q*_; therefore, it is reasonable to consider for *E*(*T*) something similar to eq 1, namely
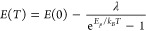
3where *E*(0) is the bandgap at 0 K, λ is a parameter related to the strength of electron–phonon interaction, and *E*_*p*_ is the average phonon energy.^36,37^Equation 3 was fitted to the direct energies extracted from eq 2 (parameter *hν*_*x*_), and this is shown in Figure 4d as the red solid line. The parameters obtained after fitting eq 3 to both indirect (absorption, Figure 3d) and direct (reflectance, Figure 4d) bandgaps are summarized in Table 2.

**Table 2:** Parameters Related to the Temperature Dependence of Energies, Obtained by Fitting Eq 3 to the Acquired Energies

Method	*E*(0) [eV]	Parameters
Absorption (IND)	2.985(1)	λ = 112 ± 5 meV, *E*_*p*_ = 8 ± 1 meV
Reflectance (FX-like)	3.040(1)	λ = 106 ± 14 meV, *E*_*p*_ = 9 ± 1 meV

It was found that the reflectivity resonance shifts from 3.028 eV at 40 K to 2.792 eV at 300 K, yielding Δ*E*_*d*_ = 0.236 eV. Such values for Δ*E*_*d*_ and Δ*E*_*i*_ (0.323 eV) are much larger than the bandgap change for other van der Waals crystals, such as MoS_2_, MoSe_2_, WS_2_, and WSe_2_, which is approximately 0.07, 0.08, 0.09, and 0.08 eV, respectively, between 50 and 300 K^38,39^.

**Figure 5:**
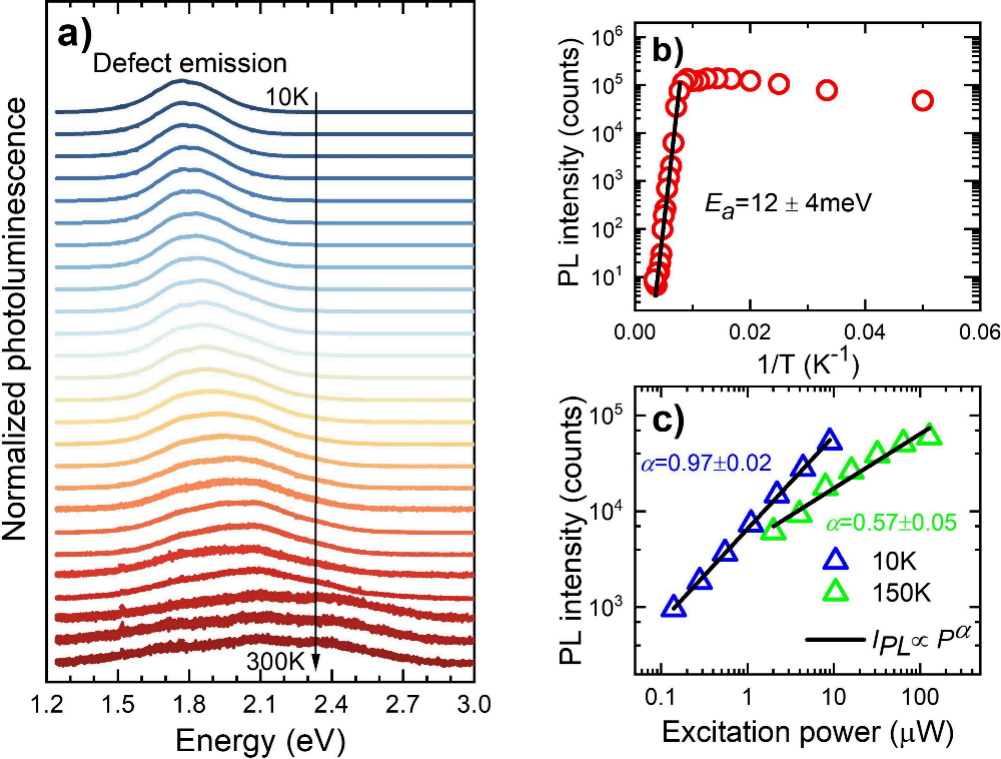
a) Normalized photoluminescence of HgPS_3_. Spectra are shifted vertically for better visualization. b) Arrhenius plot for extraction of the activation energy for defect-related emission. c) Maximum PL intensity versus excitation power (log–log plot) at 10 (blue triangles) and 150 K (green triangles).

Figure 5a shows the normalized PL spectra measured in the 10–300 K temperature range for the HgPS_3_ crystal. According to the previous discussion, this emission is attributed to defects. In general, as the temperature increases, the PL peak broadens and shifts to higher energies and its intensity decreases. The analysis of the PL peak intensity is presented in Figure 5b. Up to approximately 100 K, the PL intensity increases with increasing temperature (negative thermal quenching), which can be observed in the case of defect-related emissions for some semiconductors.^40−42^ Above this temperature, the intensity decreases, and at room temperature, it is weaker by 4 orders of magnitude. The activation energy (*E*_*a*_) calculated from the Arrhenius formula , where *I*_0_ is the PL intensity before the thermal quenching, is 12 ± 4 meV. Assuming that the character of the defect-related emission at low temperature is excitonic, the obtained activation energy can be attributed to the average exciton binding energy at the defect. The excitonic character of low-temperature emission is confirmed by power-dependent PL measurements; see Figure 5c. The PL intensity (*I*_*PL*_) increases with the excitation power (*P*) as *I*_*PL*_ ∝ *P*^α^. At 10 K, α = 0.97, which is close to 1, which is typical for excitonic emission.^43^ At 150 K, α = 0.57, which means that the excitonic character of this emission is lost. It is also worth mentioning that the intensity of PL changed with the place on the sample, but its nature remained the same (defective). Changes in PL intensity with position on the sample are attributed to different defect concentrations.

### 3.3 Electronic Band Structure

**Figure 6:**
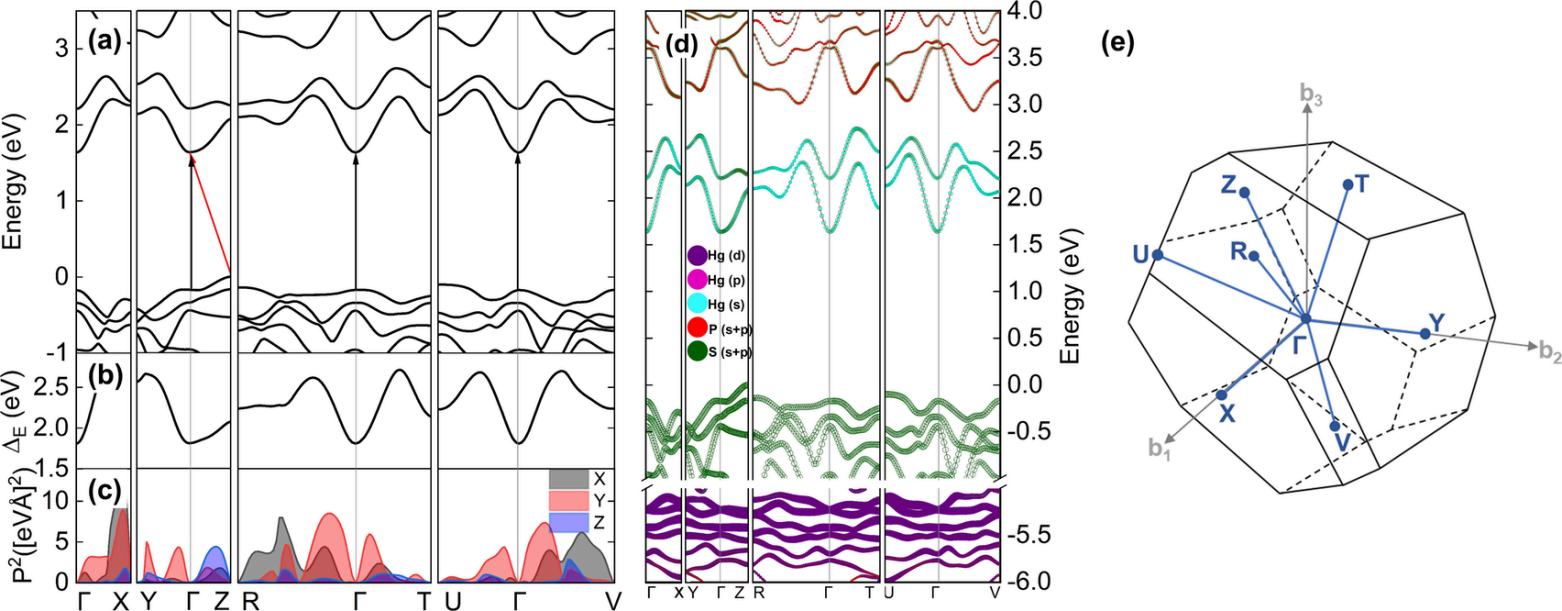
a) Electronic band structure calculated with PBE+D3+SO. The red arrow indicates the lowest energy indirect transition (*Z*–Γ), and the black arrows indicate direct transitions at the Γ k-point. b) Energy difference between valence band and conduction band which means an energy gap in the path of k points and c) the corresponding oscillator strengths of the transitions (*x*, *y*, *z* polarization components). d) Electronic structure presented in a larger energy range with a projection to specific atomic states. e) First Brillouin zone of HgPS_3_.

**Figure 7:**
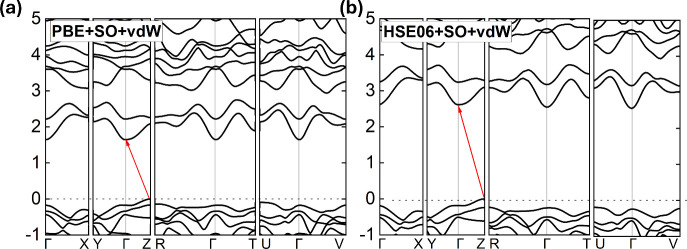
Electronic band structure of HgPS_3_ calculated with a) PBE+D3+SO and b) HSE06+SO+vdW. The red arrows indicate the lowest energy indirect transition (*Z*–Γ).

Figure 6 shows the results of PBE+D3+SO calculations for bulk HgPS_3_ on the k-path marked in blue in Figure 6e. As can be seen in Figure 6a, the fundamental transition for this system is indirect between the *Z* point in the valence band maximum (VBM) and the Γ point in the conduction band minimum (CBM), as indicated by the red arrow, but the direct transition (black arrows) at the Γ point is also energetically close: these are 1.637 and 1.809 eV, respectively. These values are lower than the experimental ones due to complications such as self-interaction errors. Therefore, we compared these results to calculations with the HSE06+SO+vDW hybrid functional, which is known to reproduce experimental gap values better than PBE. For HSE06 calculations, the mentioned values of the indirect and direct gaps are 2.613 and 2.864 eV, respectively. A comparison between the band structures obtained from both methods can be seen in Figure 7, and the increase in bandgap is evident. From Figure 6b it follows that this transition is a direct transition with the lowest energy, but it is forbidden by the selection rules (see Figure 6c). We observe the same phenomenon—zero transition oscillator strengths—for all optical transitions at high-symmetry k-points between the conduction and valence bands. However, transitions around the Γ point have a large oscillator strength polarized mainly in the plane of the layer, which means that direct transitions with similar energies will be detected by absorption-like experiments. Direct transition oscillator strength dependencies for higher energy optical transitions, along with a more extensive analysis, can be found in Figure S10. As can be seen in Figure 6d, the electronic structure itself is very rich. The d states of mercury are located deep in the valence band between −6 and −5 eV, unlike the valence states close to the energy gap, which are composed of the p states of sulfur. The two lowest energy conduction bands consist of the s states from Hg, which strongly hybridize with the p states from S. After these two bands, there is a small energy gap of several hundred meV, after which there are states composed of the s + p states of phosphorus and again sulfur.

**Figure 8:**
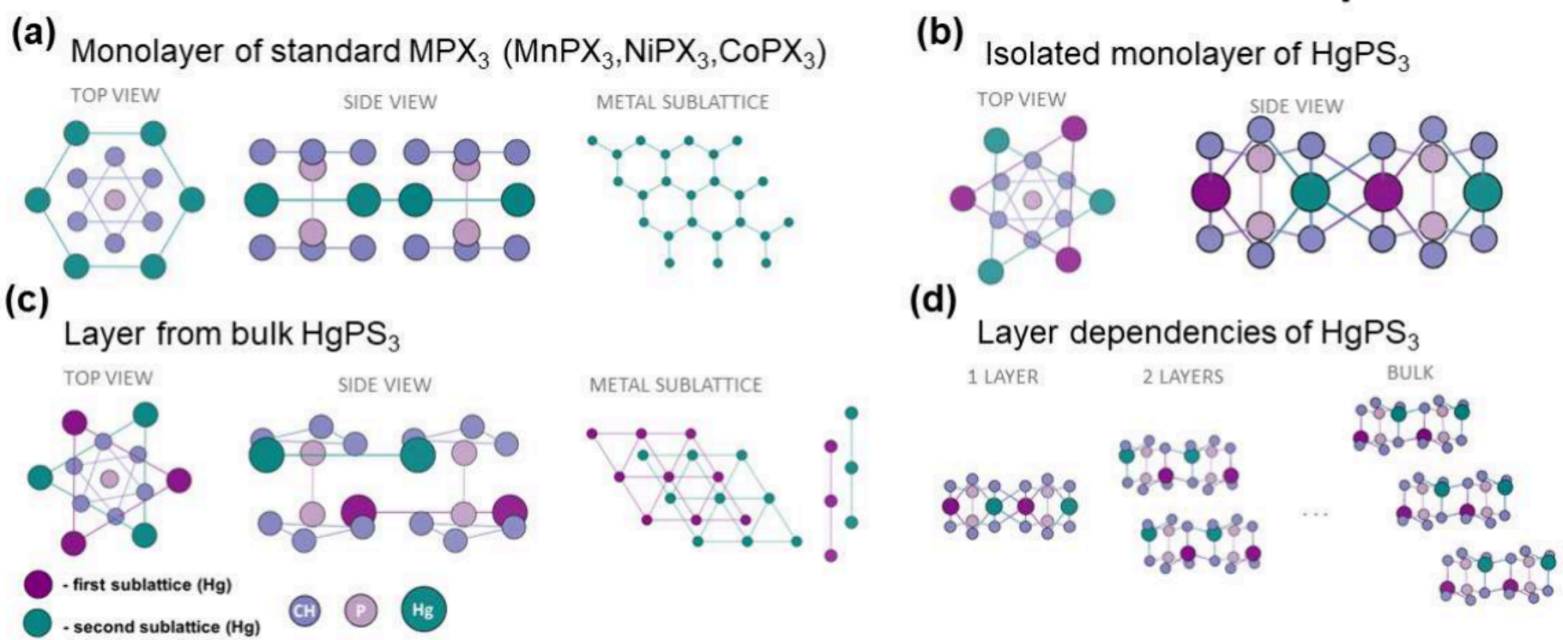
a) Side and top view (and hexagonal arrangement of metal atoms) of a typical structure of MPX_3_ crystals for monolayers. b) Structure of a monolayer taken directly from a bulk crystal. In this case, instead of a hexagonal metal lattice, we observe two sublattices. The structure of the isolated monolayer shown in c) is similar to the layer of typical crystals but differs in the arrangement of the chalcogen. d) Dependence of the geometric structure on the number of layers.

Our theoretical calculations showed that an isolated monolayer subjected to a full geometric structure optimization changes the arrangement of the metal sublattice (see Figure 8b) from two shifted trigonal sublattices of mercury atoms to a hexagonal sublattice on the same plane, obtaining a structure very similar to that of other MPX_3_ crystals (Figure 8a, differing only in the distortion of the P_2_S_6_ bipyramids). As can be seen in Figure 8c,d, from just the two-layer system, the internal structure of the layer reorganizes into a structure with two metal sublattices, just like in a bulk crystal.

**Figure 9:**
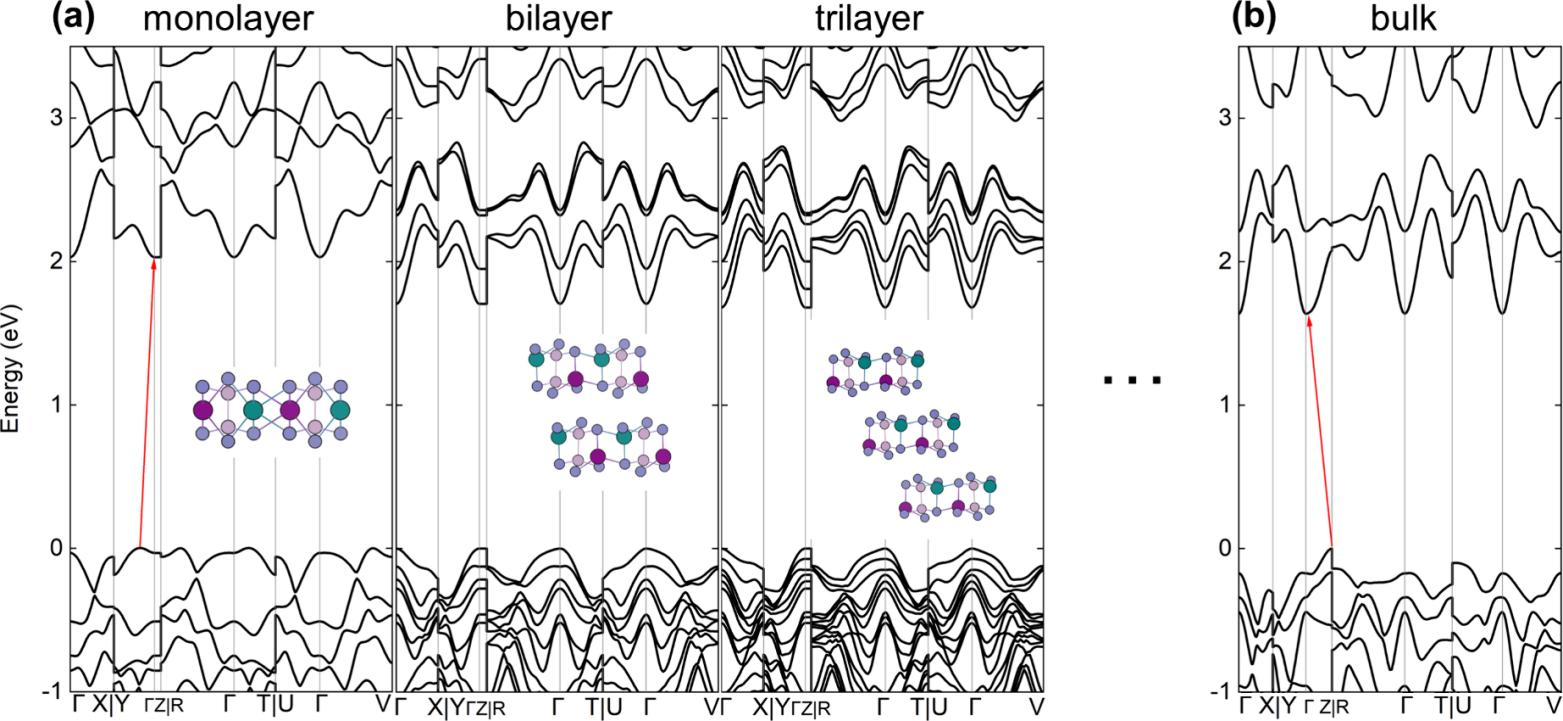
a) Electronic structure of single layers (mono-, bi-, and tri-layer) computed using PBE+D3+SO with insets showing the side view of the geometric structure. Note that a monolayer has a different structure than a layer in a multilayer system. b) Electronic structure of the bulk material. The red arrows mark the lowest energy optical transitions.

Figure 9 shows the electronic structures of the mono-, bi-, and trilayer systems and the bulk crystal. For comparison purposes, all the presented structures were calculated on the same k-point path; however, the three-dimensional Brillouin zone reduces to two-dimensional in the case of layers, which is why we observe a flat dispersion in the Γ–*Z* direction. Due to the change in the geometric structure, the band structure of the monolayer is qualitatively different from thin films and the bulk material. In this case, the fundamental transition is indirect from the point between the *Y*–Γ high-symmetry points in the valence band to the Γ point in the conduction band. In a bulk material, this transition is indirect between the Γ point and the *Z* point (reduced coordinates: 0; 0; 0.5), which is why multilayers with the same structure have a direct band gap due to the *Z* point of the three-dimensional Brillouin zone folding into the Γ point in the two-dimensional one. The intensities of a fundamental direct transition, given as  in which  is the light polarization (pol.) and  is the matrix element between CBM and VBM, for bilayer and trilayer are relatively large [(0.37; 0.07; 1.30) (eV Å)^2^ and (0.68; 0.06; 1.14) (eV Å)^2^, respectively] but are polarized mainly in the direction perpendicular to the layer. To sum up, it is possible that although a single layer or a bulk crystal does not show photoluminescent properties, thin layers, such as bilayer, may exhibit them.

### 3.4 Exfoliation of HgPS_3_

In general, like TMDs, TMPTs are lamellar materials whose layers are held together by weak van der Waals interactions, enabling mechanical exfoliation via the “Scotch tape method”, similar to how graphene was first obtained.^5^ The SEM image shown in Figure 1c confirms the layered structure of HgPS_3_. Nevertheless, some factors can hinder its exfoliation. The relatively big atomic mass of mercury (200.59 u) leads to a larger cationic radius and P–P bond length, which combined result in a thicker layer.^44^ Moreover, the unusual crystal structure, along with the presence of intrinsic defects, as confirmed by photoluminescence measurements, further complicate the process.

Our theoretical calculations, carried out according to the method proposed by Jung,^45^ showed that the exfoliation energy of HgPS_3_ equals 15.4 meV/Å, and the similarly calculated energy for MoS_2_ is 25.1 meV/Å, reflecting very well the literature values (e.g., 21.6 meV/Å for DF2-C09 calculations).^46^ According to the nomenclature adopted in the work of N. Mounet et al.,^46^ both these compounds qualify as “easy exfoliable” materials. This is typical for materials from the MPX_3_ group, for which van der Waals bonding forces are usually weak.^18^ The interlayer component of the contribution of van der Waals forces to the energy, without taking into account the interactions within the layer, is approximately 0.19 eV/atom in the case of HgPS_3_.

**Figure 10:**
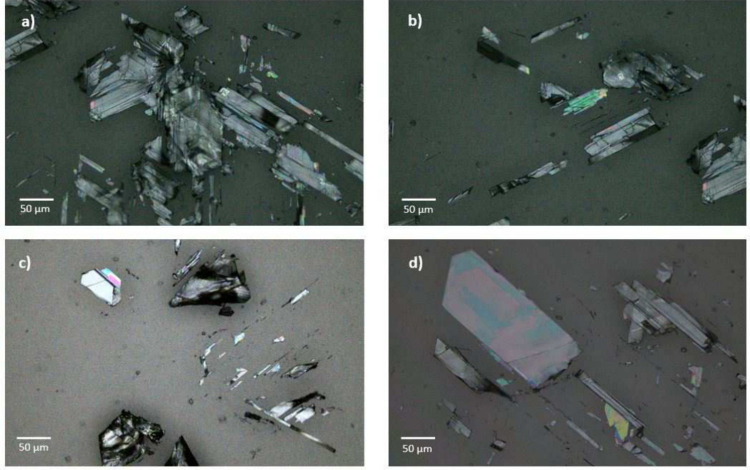
Optical images of mechanically exfoliated HgPS_3_ flakes on PDMS taken in reflection illumination mode with 10× magnification.

To verify this experimentally, HgPS_3_ flakes were mechanically exfoliated from bulk crystals with Low Tack tape and then transferred onto a polydimethylsiloxane (PDMS) substrate. Figure 10 shows the exfoliated flakes. One can clearly see that after many steps of exfoliation there are still many bulky flakes (gray parts), and a small percentage becomes thinner (colorful flakes); therefore, the yield of few-layer flakes is relatively low when compared to the exfoliation of MoS_2_, for instance, under the same conditions. This is supported by the atomic-force microscopy (AFM) measurements that provided flake thickness in the range 30–250 nm (Figure S9), which is much thicker than a monolayer of MoS_2_ or NiPS_3_: 0.6–0.8 nm and ≈1 nm, respectively.^7,47^ To conduct a comparative analysis, exfoliation of MoS_2_ crystals was performed, specifically examining the exfoliation yield concerning few-layer flakes for both compounds. Optical images of exfoliated MoS_2_ can be seen in Figure S6a and differential reflectance was performed on a flake to check its monolayer character (Figure S6b). The calculated values for exfoliation energy suggest that HgPS_3_ should be easier to exfoliate than MoS_2_, but this has not been experimentally observed. It suggests that solely the exfoliation energy is not a key factor for determining the ease of mechanical exfoliation, and other aspects are also responsible for the low yield of thin HgPS_3_ flakes, such as structural and mechanical anisotropies.^48^

## 4 Conclusions

In summary, the electronic band structure, structural and optical properties of HgPS_3_ crystal and layers were studied experimentally and theoretically. It was concluded that this crystal has an indirect gap of 2.68 eV at 300 K and a direct gap at the Γ point of BZ approximately 50 meV above the indirect gap. The optical transition at the Γ point is forbidden due to the selection rules, but near the Γ point the direct optical transitions are allowed and therefore they are visible in reflectance spectra. The indirect absorption can be observed in the absorption spectra, occurring at approximately 60–120 meV below the direct transitions observed in reflectance, throughout the range 40–300 K. The temperature dependence of indirect and direct gaps has been determined, respectively, from absorption and reflectance measurements. Both indirect and direct gaps show strong red shifts of 0.323 and 0.236 eV, respectively, with increasing temperature, which are much higher than those for other van der Waals crystals in the same temperature range. In the PL spectra, no near-band gap emission was observed due to the indirect gap, but defect-related emission was clearly visible ≈1 eV below the absorption edge. It was found that the electronic band structure of HgPS_3_ undergoes significant changes when the crystal thickness is reduced to tri- and bilayers, resulting in a direct bandgap. Interestingly, in the monolayer regime, the fundamental transition is indirect as in the case of the bulk crystal, therefore differing from TMDs and α-PbO. Finally, mechanical exfoliation of HgPS_3_ crystals was proven to be experimentally more challenging than that of MoS_2_ crystals, despite its smaller exfoliation energy.

## Supporting Information Available

XRD, EDS, SEM/EDS, XPS, and FTIR spectra of HgPS_3_; mechanical exfoliation and differential reflectance of MoS_2_ for the purpose of comparison with the exfoliation yield of HgPS_3_; Raman spectroscopy, AFM, and DFT calculations of oscillator strength and dielectric function (PDF)
