# Supplementary material for: Electronic Band Structure and Optical Properties of HgPS3 Crystal and Layers
Source: J Phys Chem C Nanomater Interfaces. 2024 May 24;128(22):9270–80. doi: 10.1021/acs.jpcc.4c00562 (PMC11163980; doi:10.1021/acs.jpcc.4c00562)
Supplement: Supplementary file 1 — jp4c00562_si_001.pdf [file jp4c00562_si_001.pdf]

Electronic Band Structure and Optical Properties of $HgPS_3$ Crystal and Layers

Beatriz de Simoni*[1], Miłosz Rybak[1], Nikolas Antonatos[1,2], Artur P. Herman[1], Karolina Ciesiołkiewicz[1], Agata K. Tołłoczko[1], Maciej Peter[1], Adrianna Piejko[3], Kseniia Mosina[2], Zdeněk Sofer[2], Robert Kudrawiec**[1]

[1]Department of Semiconductor Materials Engineering, Wroclaw University of Science and Technology, Wybrzeże Wyspiańskiego 27, 50-370 Wrocław, Poland
[2]Department of Inorganic Chemistry, University of Chemistry and Technology, Technická 5, 166 28 Prague 6 – Dejvice, Czech Republic Czech Republic
[3] Department of Nanometrology, Wroclaw University of Science and Technology, Janiszewskiego 11/17,50-370 Wrocław, Poland
* beatriz.desimoni@pwr.edu.pl
** robert.kudrawiec@pwr.edu.pl

Supplementary Information

Structural Characterization

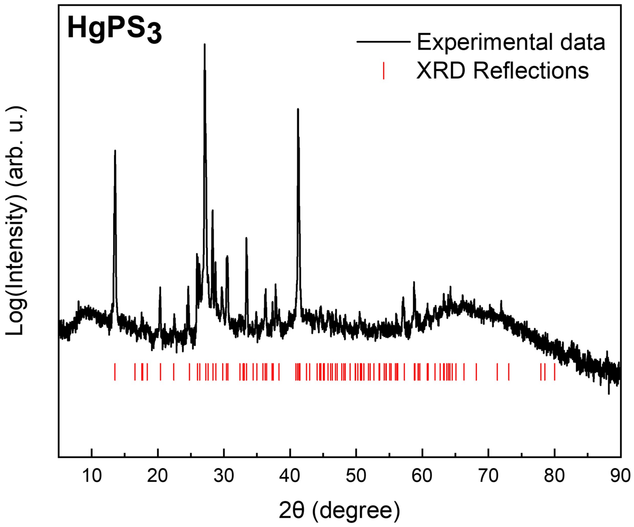

Figure S1: The XRD pattern plotted on logarithmic scale with all the simulated XRD reflections.

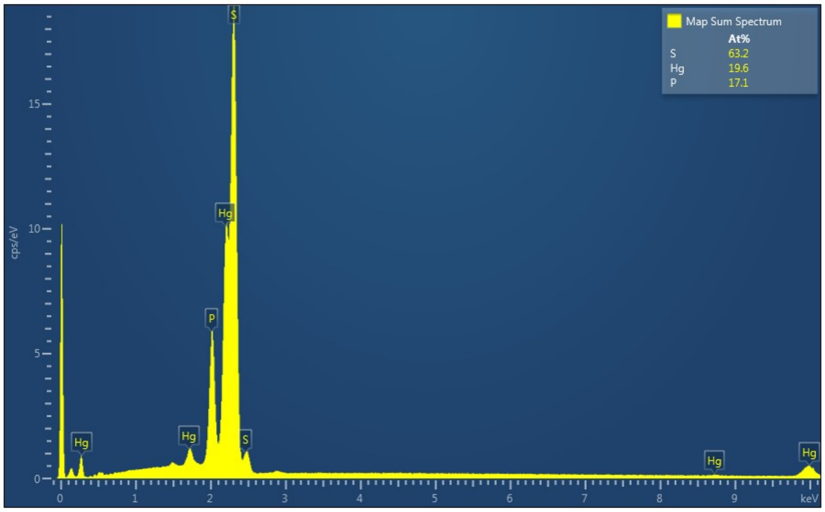

Figure S2: SEM image of $HgPS_3$ with the corresponding EDS elemental maps of mercury, phosphorus, and sulphur.

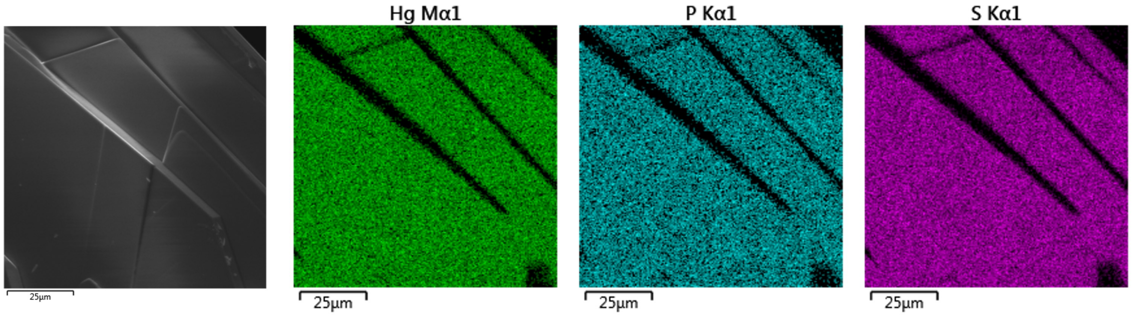

Figure S3: SEM/EDS map spectrum of $HgPS_3$.

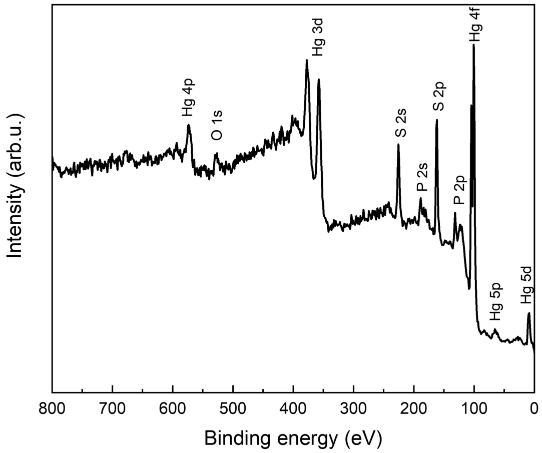

Figure S4: Wide-survey XPS spectrum of $HgPS_3$.

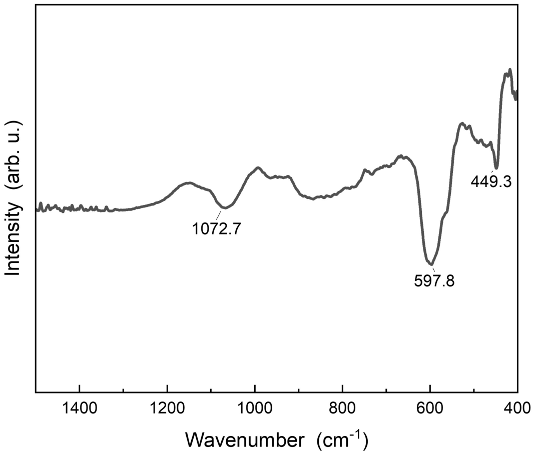

Figure S5: FT-IR spectrum of $HgPS_3$.

Exfoliation of $HgPS_3$

Figure S6a shows how nearly transparent a monolayer is when the microscope is on reflection mode and with its diaphragm open. In the case of few-layers (e.g. 1, 2, 3 layers) one expects very low optical contrast, in such a way that the color of the flakes will be very similar to that of the substrate. One way to assure that the flake is a monolayer is by collecting differential reflectance spectrum between the flake and the substrate (Figure S6b) and check the spectral positions of the A, B and C excitons, which are well established for MoS_2 as well as $MoSe_2$, WS_2 and WSe_2. In the case of monolayer MoS_2 the narrow A exciton is located expected at approximately 1.92 eV, while B is at around 2.03 and the broad C exciton is expected at around 2.81 eV, and these values can vary 10-15 meV from flake to flake [1]. The flake indicated by the arrow is therefore a monolayer.

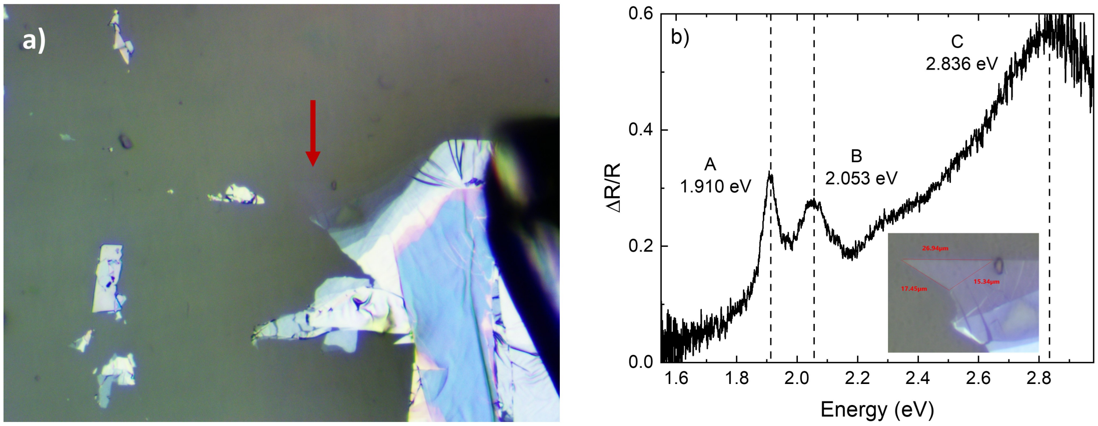

Figure S6: a) Optical images of mechanically exfoliated MoS_2 flakes transferred to SiO_2/Si substrate with 20x magnification. Red arrow indicates a monolayer candidate. b) Differential reflectance from the flake indicated by the arrow, which confirms that it is a monolayer. Dashed lines highlight spectral position of excitons A, B, and C. Inset shows an image of the monolayer with 50x magnification and closed diaphragm for better visualization. Red lines indicate the approximate flake dimensions: 27x17x15 μm.

Raman Spectroscopy

To the best of our knowledge, there is no literature regarding the Raman modes of $HgPS_3$. Therefore, in this study we present an analysis of its vibrational modes by combining experimental data and DFT calculations. Figure S7a shows Raman spectra of bulk $HgPS_3$ from 10 to 300 K, and 12 peaks can be identified at low temperature, labeled P1 to P12 from low to high frequency, respectively and indicated by the arrows. In general, all of them undergo a slight shift to lower frequencies as the temperature increases (Figure S7b), apart from the usual decrease in intensity. Nevertheless, 7 out of the 12 peaks are still visible at 300 K. P2, P5, P11 and P12 vanish at around 200 K, while P7 vanishes at 280 K, as shows Figure S7b. Peak frequencies were extracted by Lorentz fits. No peak splitting is observed. These results suggest that the compound does not undergo structural phase transition in this temperature range. Raman spectra of exfoliated crystals of $HgPS_3$ are presented in Figure S7c. Three flakes were investigated at room temperature, and seven peaks can be observed: P1, P3, P4, P6, P8, P9, and P10, in excellent agreement with the spectrum of the bulk crystal at the same temperature. No significant difference between bulk and exfoliated $HgPS_3$ phonon modes was noticed. The exfoliated flakes were transferred onto a Si/SiO$_2$ substrate, and the intense peak at around 515 cm^{-1} is due to the substrate.

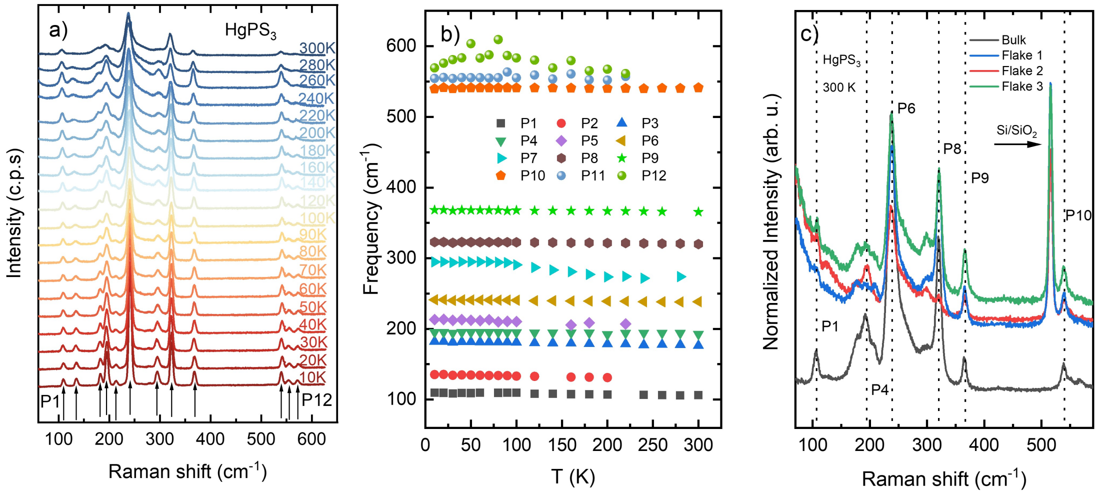

Figure S7: a) Raman spectra of bulk $HgPS_3$ from 10 to 300 K. Spectra has been shifted vertically for better visualization. b) Temperature dependence of the frequency of the 12 peaks marked in a) by arrows. c) Raman spectra of exfoliated $HgPS_3$ flakes at 300 K. Peaks are labelled according to a).

Phonon calculations at the Γ point of the Brillouin Zone yielded 27 modes, with irreducible representation $\Gamma = 15A_g + 12A_u$, labeled Ag1 to Ag15 and Au1 to Au12, and marked by black and red vertical lines, respectively, in Figure S8. The main feature is at $\approx$ 241 cm^{-1} with a corresponding calculated phonon mode at $\approx$ 244 cm^{-1}. According to [2], only A_g modes are Raman-active. The corresponding calculated phonon frequencies are given in Table S1, together with the frequencies of the 12 peaks experimentally observed at 10 K. The fact that 12 peaks were observed experimentally might be related to the setup configuration: some peaks might only be visible with parallel scattering configuration (the conventional one, used by us, in which incident and scattered light are parallel to each other), while others might only be accessed in cross-scattering configuration.

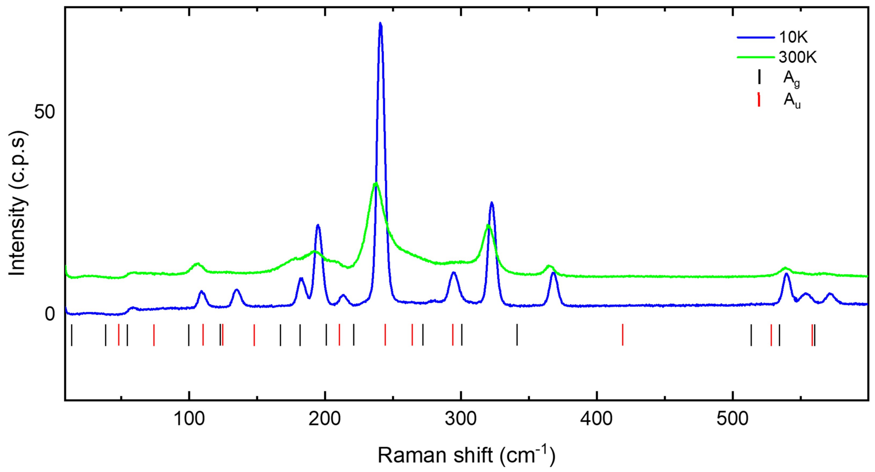

Figure S8: Raman spectrum of $HgPS_3$ at 10 K (blue) and 300 K (green), along with calculated phonon modes A_g (black) and A_u (red).

Mode	Calculated Frequency (cm^{-1})	Peak	Experimental Frequency @10K (cm^{-1})
Ag1	559.424	P12	569.359
Au1	558.954	P11	554.379
Ag2	534.096	P10	539.732
Au2	528.699		
Ag3	513.052		
Au3	418.814	P9	368.219
Ag4	341.010	P8	322.905
Ag5	300.717		
Au4	293.593	P7	294.774
Ag6	272.007		
Au5	264.453		
Au6	244.093	P6	241.152
Ag7	221.833		
Au7	210.728	P5	213.139
Ag8	201.886	P4	195.223
Ag9	181.347	P3	182.297
Ag10	167.036		
Au8	147.930	P2	135.421
Au9	124.061		
Ag11	123.204		
Au10	110.236	P1	109.652
Ag12	99.231		
Au11	74.062		
Ag13	54.427		
Au12	48.543		
Ag14	38.353		
Ag15	13.881		

Table S1: Calculated phonon modes, the 12 experimentally observed peaks from bulk $HgPS_3$ at 10 K and corresponding frequencies.

Atomic Force Microscopy

Atomic force microscopy (AFM) revealed that the thickness of the exfoliated flakes varies from 30 to 250 nm. The measurements were carried out using a NanoAndMore ARROW-Cont Pt probe with a force constant of ≈ 0.2 N/m and a resonant frequency of ≈ 15 kHz in contact mode. AFM results can be seen in Figures S9 and Table S2. Some investigate flakes such as flake 1 from substrate 1 (Figure 9a) and flake 1 from substrate 2 (Figure 9c) clearly show the layered structure of $HgPS_3$, already confirmed by SEM.

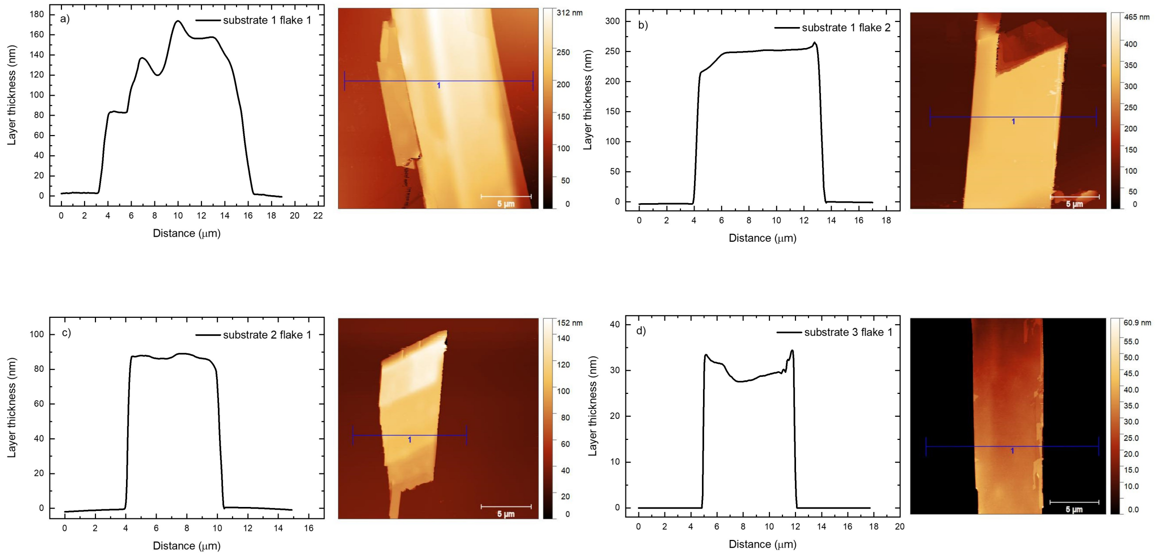

Figure S9: (a-d) Height profiles of four different flakes, along with their AFM image. Scale bars (white) is 5μm and blue bars show the direction that the measurements were made.

	Layer thickness (nm)
flake 1 substrate 1	160
flake 2 substrate 1	250
flake 1 substrate 2	85
flake 1 substrate 3	30

Table S2: Thicknesses of the exfoliated flakes shown in Figure S9.

DFT Calculations

Oscillator Strength

Figure S10a shows the energy difference of the valence (3 bands) and conduction (2 bands) bands closest to the energy gap. The blue circles indicate the four lowest energy direct transitions at high symmetry points or local minima that have nonzero transition oscillator strengths (See Figure S10b, which presents specific values of these quantities for the x, y and z polarization components). S10c collects information from the previous figures. The size of the circle indicates the sum of the strength components of the transition oscillators, and the colors indicate the transition between specific bands. Higher energy optical transitions are possible at the Γ point, approximately at a few hundreds meV above the absorption edge.

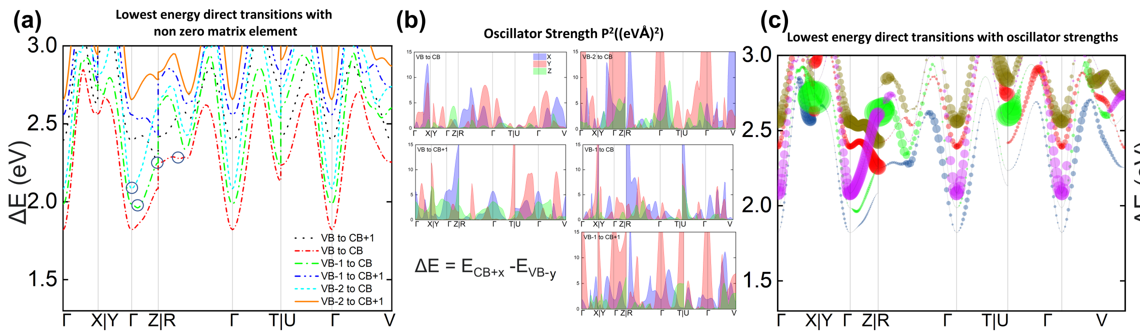

Figure S10: a) Lowest energy direct transitions with non-zero matrix elements. b) Oscillator strength of each of the transitions for the x, y and z polarization components. c) combines a) and b): size of the circle indicates the sum of the oscillator strength, and the colors indicate the transition between specific bands.

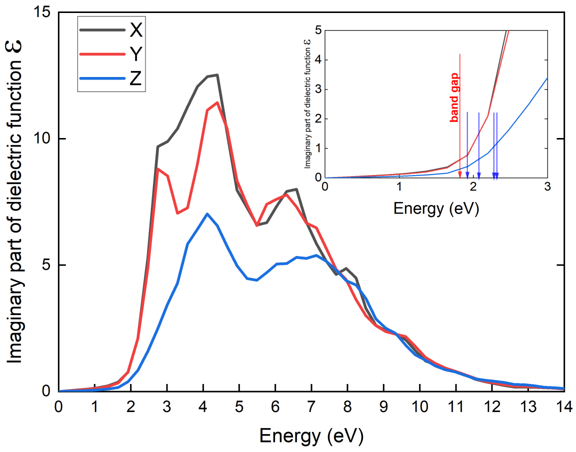

Figure S11: Single particle dielectric function of $HgPS_3$ obtained by density-functional perturbation theory (DFPT). The inset includes a close-up of the edge with arrows marking the band gap, and the lowest optical transitions with non-zero oscillator strength presented in Figure S10a.

References

[1] Niu Y.; Gonzalez-Abad S.; Frisenda R.; Marauhn P.; Drüppel M.; Gant P.; Schmidt R.; Taghavi N. S.; Barcons D.; Molina-Mendoza A. J. et al. Thickness-Dependent Differential Reflectance Spectra of Monolayer and Few-Layer MoS_2, $MoSe_2$, WS_2 and WSe_2. *Nanomaterials* **2018**, *8*, 725.

[2] Oliva R.; Ritov E.; Horani F.; Etxebarria I.; Budniak A. K.; Amouyal Y.; Lifshitz E.; Guennou M. Lattice Dynamics and In-Plane Antiferromagnetism in $Mn_xZn_{1-x}PS_3$ Across the Entire Composition Range. *Phys. Rev. B* **2023**, *107*, 104415.
